# Association between triglyceride–glucose index and gallstone prevalence in American adults: A cross-sectional analysis of NHANES 2017 to 2020 data

**DOI:** 10.1097/MD.0000000000045991

**Published:** 2025-11-14

**Authors:** Jianan Tu, Xinlong Li

**Affiliations:** a Nursing Department, Sir Run Run Shaw Hospital, Zhejiang University School of Medicine, Shangcheng District, Hangzhou, Zhejiang Province, China; b Sir Run Run Shaw Hospital, Zhejiang University School of Medicine, Shangcheng District, Hangzhou, Zhejiang Province, China.

**Keywords:** cross-sectional study, gallstones, insulin resistance, NHANES, TyG index

## Abstract

To examine the association between the triglyceride–glucose (TyG) index, a surrogate marker of insulin resistance, and gallstone prevalence among U.S. adults. Data from the 2017 to 2020 National Health and Nutrition Examination Survey were utilized. Adults aged ≥ 20 years with complete information on gallstone history, TyG index, and covariates were included (n = 7806). Multivariable logistic regression, subgroup analyses, and smooth curve fitting were employed to assess the TyG–gallstone association. Among 7806 participants, 836 (10.7%) reported gallstones. After full adjustment, each one-unit increase in the TyG index was associated with higher odds of gallstones (odds ratio = 1.51, 95% confidence interval: 1.33–1.71). A nonlinear dose–response relationship was observed, with a significant positive association below a TyG threshold of 8.97 (odds ratio = 2.36, 95% confidence interval: 1.85–3.03). Subgroup analyses demonstrated consistent associations across sex, age, race/ethnicity, diabetes, and hypertension strata (all interaction *P* > .05). A higher TyG index is independently associated with increased gallstone prevalence in U.S. adults. These findings support a potential role of insulin resistance in gallstone pathogenesis and warrant longitudinal studies to establish causality.

## 1. Introduction

Gallstone disease is a common hepatobiliary condition with substantial clinical and economic impact worldwide.^[1]^ In the United States, gallstones are a leading cause of gastrointestinal-related hospitalizations and cholecystectomy, contributing to significant morbidity through biliary colic, acute cholecystitis, pancreatitis, and cholangitis.^[2,3]^ Gallstones impose substantial healthcare costs-estimated at roughly $6 billion annually (and are associated with an elevated risk of gallbladder cancer).^[4]^ Identifying modifiable risk factors and reliable biomarkers to stratify risk is therefore a clinical priority.

Insulin resistance is implicated in hepatic cholesterol overproduction, altered bile composition, and impaired gallbladder motility, all of which may favor cholesterol supersaturation and lithogenesis.^[5,6]^ While clamp-based techniques are the gold standard for quantifying insulin resistance (IR), they are impractical in population studies.^[7]^ Surrogate indices derived from routine laboratory tests have therefore gained traction. The triglyceride–glucose index (TyG), calculated from fasting triglycerides and glucose, has shown good performance for identifying IR and cardiometabolic risk in diverse populations.^[8–10]^ Nevertheless, the relationship between TyG and gallstone disease has not been well characterized, and evidence from U.S. adults, particularly using nationally representative data, is limited.

Therefore, we investigated the correlation between the TyG indicator and the probability of gallstones by analyzing data from the 2017 to 2020 National Health and Nutrition Examination Survey (NHANES) on American male participants.

## 2. Materials and methods

### 2.1. Study design and participants

The study utilized publicly available data from the NHANES, a nationally representative cross-sectional program administered by the U.S. Centers for Disease Control and Prevention. NHANES is conducted in 2-year cycles, each sampling approximately 10,000 noninstitutionalized U.S. residents. Due to the COVID-19 pandemic, the 2019 to 2020 cycle achieved only partial data collection (approximately 60% of the target sample size). We combined data from the 2017 to 2020 cycles because the gallstone questionnaire was consistently administered during these years.

**Figure 1.**
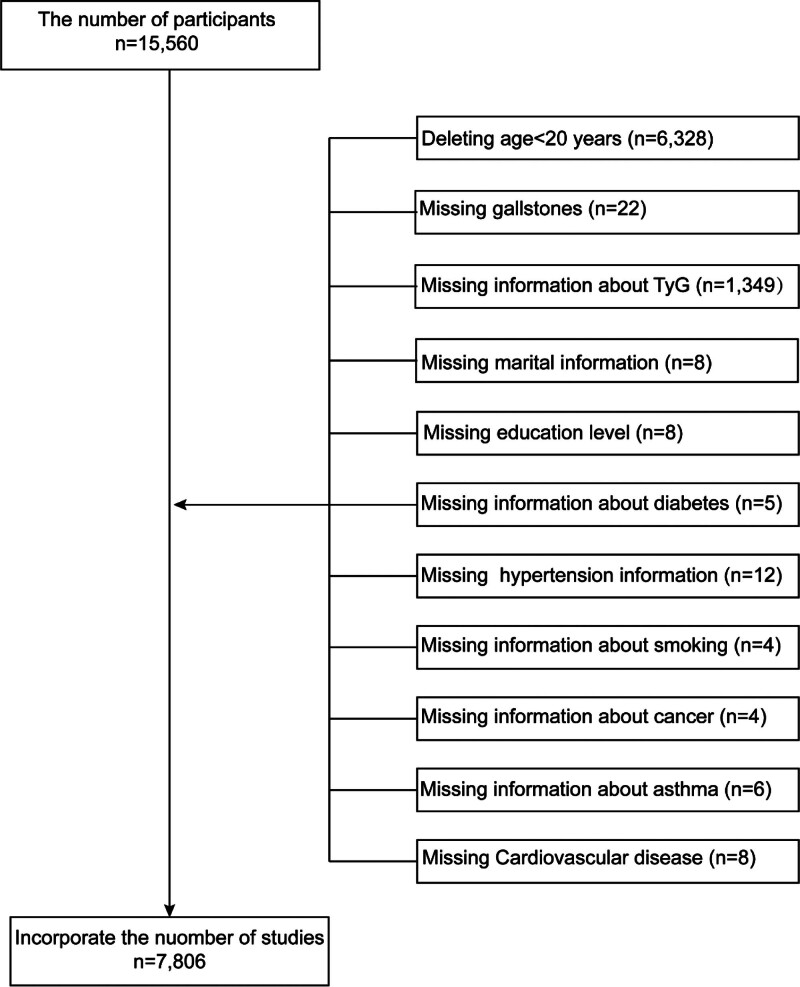
Flowchart of the sample selection from National Health and Nutrition Examination Survey (NHANES) 2017 to 2020.

From 15,560 participants initially identified, we excluded 6328 individuals aged <20 years; 22 without completed gallstone questionnaires; 1349 with missing TyG index data; and 55 with missing covariate data (marital status, educational attainment, hypertension, diabetes, smoking, cancer, asthma, or cardiovascular disease). The final analytic sample comprised 7806 adults. The participant selection process is summarized in Figure 1.

### 2.2. Definition of gallstones

Gallstone status was ascertained via questionnaire using the item: “Has DR ever said you have gallstones.” Participants answering “yes” were classified as having gallstones; those answering “no” were classified as not having gallstones.

### 2.3. Calculation of TyG

The TyG index was the primary exposure. Following prior studies,^[11]^ TyG was calculated as: TyG = Ln (fasting triglycerides [mg/dL] * fasting glucose [mg/dL]/2).^[11]^ Fasting triglycerides and glucose were measured enzymatically on automated analyzers. Serum triglycerides were assayed using the Roche Modular P (Roche Diagnostics, Indianapolis) and Roche Cobas 6000 chemistry analyzers (Roche Diagnostics, Indianapolis).

### 2.4. Identification of covariates

Based on prior literature,^[12,13]^ we included potential confounders: sex, age, race/ethnicity, education, poverty income ratio, body mass index, weight, marital status (married/living with partner vs single), alcohol consumption (current drinking vs not), physical activity (vigorous, moderate, or less than moderate), smoking status, hypertension, diabetes, asthma, cancer, cardiovascular disease (CVD), and dietary intake (energy, fat, sugar, and water). Dietary variables were derived from two 24-hour recalls (2017–2020); we used the average intake across recalls. Diabetes was defined by a “yes” response to “Has a doctor ever told you that you have diabetes?” Hypertension was defined by a “yes” response to “Ever told you had high blood pressure.” CVD was defined by self-reported physician-diagnosed heart failure, angina, coronary heart disease, myocardial infarction, or stroke. Detailed measurement procedures are publicly available at www.cdc.gov/nchs/nhanes/.

### 2.5. Statistical analyses

Analyses followed Centers for Disease Control and Prevention guidance for NHANES, incorporating appropriate sampling weights to account for the complex multistage design. Continuous variables are reported as means ± standard deviations; categorical variables as percentages. We estimated odds ratios (ORs) and 95% confidence intervals (CIs) for the association between TyG and gallstones using logistic regression. Three models were specified: Model 1, unadjusted; Model 2, adjusted for sex, age, race/ethnicity, education, and marital status; Model 3, fully adjusted for all covariates listed above. Additionally, we investigated the relationship between gallstones and the TyG index in more detail using the smooth curve fitting, inflection points were identified using likelihood ratio tests. Statistical significance was defined as 2-sided *P* < .05. Analyses were conducted using Empower (www.empowerstats.com; X&Y Solutions, Inc., Boston).

## 3. Results

### 3.1. Baseline characteristics of participants

The analysis included 7806 adults, of whom 836 had gallstones and 6970 did not, yielding a prevalence of 10.7%. The gallstone group had a higher TyG index (8.82 ± 0.62) than the non-stone group (8.64 ± 0.65) (*P* < .001). Table 1 presents the characteristics of the participants both non-stone formers and stone formers. Stone formers exhibited markedly higher TyG index and TyG-related measures (fasting glucose and triglycerides) than those non-stone formers (all *P* < .001). They also showed higher prevalences of asthma, cardiovascular disease, cancer, and current smoking, alongside lower total energy and fat intake and lower educational attainment (all *P* < .05). In contrast, age, sex, hypertension, diabetes, alcohol consumption, and several anthropometric and sociodemographic factors did not differ significantly between groups.

**Table 1:** Baselines characteristics of participants.

Characteristic	Non-stone formers (n = 6970)	Stone formers (n = 836)	*P*-value
Age (yr)	50.99 ± 17.47	50.70 ± 17.50	.652
*Gender (%*)			.247
Male	33.54–48.12	50.24	
Female	36.16–51.88	49.76	
*Race/ethnicity (%*)			.866
Mexican American	12.12	11.48	
Other Hispanic	10.37	11.36	
Non-Hispanic White	35.38	34.33	
Non-Hispanic Black	25.34	25.72	
Other race	16.79	17.11	
*Education level (%*)			<.001
Less than hight school	17.91	24.04	
Hight school	23.80	25.60	
Above hight school	58.29	50.36	
Weight (kg)	83.68 ± 23.04	84.21 ± 22.48	.275
BMI	30.04 ± 7.50	30.21 ± 7.56	.479
Waist circumference (cm)	101.10 ± 17.12	100.87 ± 16.54	.906
Energy (kcal)	2051.14 ± 841.71	1916.57 ± 772.77	<.001
Total sugar (g)	100.63 ± 62.76	102.23 ± 65.69	.330
Total fat (g)	83.64 ± 40.35	79.53 ± 38.39	.009
Total water (g)	1718.50 ± 1722.82	1650.65 ± 1735.93	.202
*PIR (%*)			.212
<1.3	24.13	25.00	
≥1.3–<3.5	34.13	32.89	
≥3.5	28.49	26.56	
Unclear	13.24	15.55	
*Marital status (%*)			.368
Cohabitation	58.05	58.37	
Solitude	22.88	21.05	
Never married	19.07	20.57	
*Physical activity (%*)			.564
Vigorous	24.09	22.49	
Moderate	23.69	24.64	
Inactive	52.22	52.87	
*Alcohol (%*)			.812
Yes	85.84	85.05	
No	8.26	8.85	
Unclear	5.90	6.10	
*Hypertension (%*)			.190
Yes	38.94	36.60	
No	61.06	63.40	
*Diabetes (%*)			.109
Yes	15.22	17.34	
No	84.78	82.66	
*Asthma (%*)			<.001
Yes	15.21	20.57	
No	84.79	79.43	
*CVD (%*)			<.001
Yes	11.09	23.33	
No	88.91	76.67	
*Cancer (%*)			<.001
Yes	9.56	17.34	
No	90.44	82.66	
*Smoked (%*)			.010
Yes	2869 (41.16%)	383 (45.81%)	
No	4101 (58.84%)	453 (54.19%)	
Glucose (mg/dL)	101.76 ± 36.56	108.92 ± 40.59	<.001
Triglyceride (mg/dL)	137.05 ± 101.04	147.51 ± 91.83	<.001
TyG index	8.64 ± 0.65	8.82 ± 0.62	<.001

CVD = cardiovascular disease, TyG = triglyceride–glucose index.

### 3.2. Logistic regression results between TyG index and gallbladder stones

Table 2 demonstrates a significant association between the TyG index and gallstone risk. In the fully adjusted model (Model 3), each one-unit increase in TyG was independently associated with 51% higher odds of gallstones (OR = 1.51, 95% CI: 1.33–1.71). When analyzed by tertiles, participants in the highest tertile had 2.28-fold greater odds compared with the lowest tertile (95% CI: 1.83–2.86), indicating a dose–response relationship.

**Table 2:** Logistic regression analysis between TyG with gallbladder stone prevalence.

Characteristic	Model 1 OR (95% CI)	Model 2 OR (95% CI)	Model 3 OR (95% CI)
TyG	1.47 (1.33–1.63) < 0.0001	1.44 (1.29–1.60) < 0.0001	1.51 (1.33–1.71) < 0.0001
*Categories*			
Tertile 1	1.0	1.0	1.0
Tertile 2	1.64 (1.35–1.99) < 0.0001	1.61 (1.33–1.96) < 0.0001	1.65 (1.31–2.07) < 0.0001
Tertile 3	2.13 (1.77–2.57) < 0.0001	2.06 (1.70–2.49) < 0.0001	2.28 (1.83–2.86) < 0.0001

Model 1 = no covariates were adjusted. Model 2 = Model 1 + age, gender, race, education level, and marital status were adjusted. Model 3 = Model 2 + BMI, weight, waist circumference, diabetes, hypertension, PIR, asthma, total water, energy, total fat, total sugar, smoked, physical activity, alcohol, cancers, and CVD were adjusted.

BMI = body mass index, CI = confidence interval, CVD = cardiovascular disease, OR = odds ratio, PIR = poverty income ratio, TyG = triglyceride–glucose index.

### 3.3. TyG index’s dose–response and threshold effect on gallbladder stone prevalence

**Figure 2.**
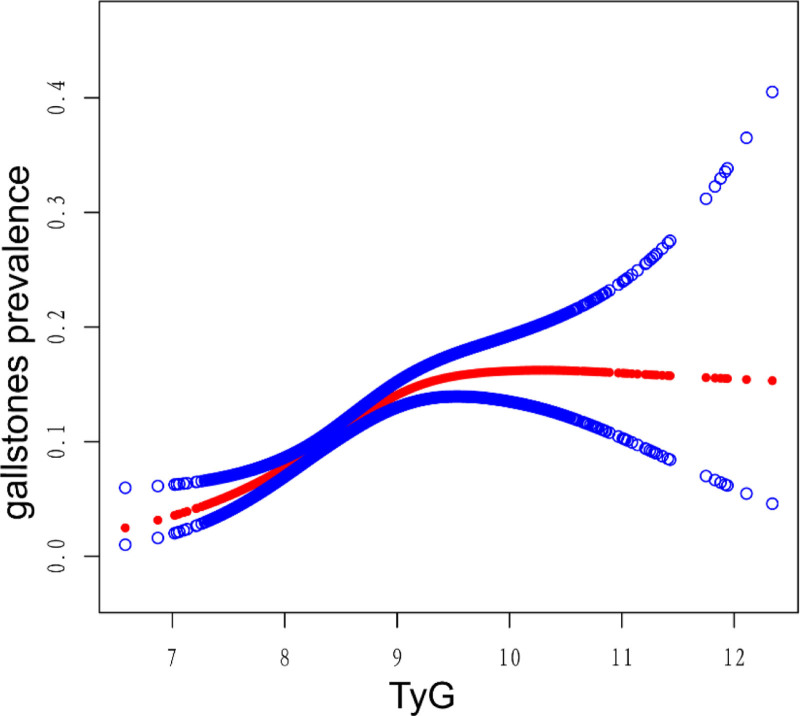
Dose–response curve between TyG index and gallstone prevalence. The solid line depicts the estimated effect; the 95% CI is the band between the upper and lower dashed lines. Models were adjusted for all covariates except the effect modifier. CI = confidence interval, TyG = triglyceride–glucose index.

We employed a smooth curve fitting to examine the relationship between TyG and gallstones. A nonlinear association was demonstrated (Fig. 2). The likelihood ratio test identified an inflection at TyG = 8.97, consistent with a saturation effect (Table 3). Below 8.97, higher TyG was strongly associated with gallstones (OR = 2.36; 95% CI: 1.85–3.03; *P* < .0001). Above 8.97, the association plateaued and was null (OR = 0.97; 95% CI: 0.75–1.25; *P* = .8029).

**Table 3:** Threshold effect analysis of triglyceride–glucose index and gallbladder stone prevalence using the 2-segment piecewise linear regression model.

Gallbladder stone	Adjusted OR (95% CI) *P* value
*TyG index*	
Inflection point	8.97
TyG index < inflection point	2.36 (1.85–3.03) <.0001
TyG index > inflection point	0.97 (0.75–1.25) .8029
Log-likelihood ratio	<.001

Adjusted for all covariates except effect modifier.

CI = confidence interval, OR = odds ratio, TyG = triglyceride–glucose index.

### 3.4. Subgroup analysis

We conducted subgroup analyses to evaluate effect modification of the TyG–gallstone association by sex, age, race/ethnicity, diabetes, and hypertension. No significant interactions were detected (all *P* for interaction > .05). The association between TyG and gallstone prevalence was therefore consistent across these strata, supporting its applicability across diverse demographic and clinical groups (Table 4).

**Table 4:** Subgroup analysis between TyG index with gallbladder stone prevalence.

Characteristic	Model 1 OR (95% CI)	Model 2 OR (95% CI)	Model 3 OR (95% CI)	*P* for interaction
*Stratified by gender*				
Male	1.49 (1.28–1.73)	1.44 (1.24–1.68)	1.48 (1.23–1.76)	.7462
Female	1.46 (1.26–1.68)	1.43 (1.24–1.65)	1.54 (1.30–1.82)	
*Stratified by age (yr*)				
<60	1.49 (1.31–1.70)	1.46 (1.27–1.66)	1.58 (1.35–1.85)	.3422
≥60	1.44 (1.21–1.70)	1.41 (1.19–1.67)	1.40 (1.15–1.71)	
*Stratified by race*				
Mexican American	1.43 (1.05–1.96)	1.41 (1.03–1.94)	1.46 (1.01–2.12)	.2190
Other Hispanic	1.75 (1.30–2.35)	1.70 (1.27–2.29)	1.82 (1.28–2.58)	
Non-Hispanic White	1.43 (1.20–1.71)	1.40 (1.17–1.67)	1.43 (1.16–1.76)	
Non-Hispanic Black	1.25 (1.02–1.53)	1.22 (0.99–1.50)	1.28 (1.01–1.64)	
Other race	1.82 (1.41–2.36)	1.78 (1.38–2.31)	1.94 (1.43–2.64)	
*Diabetes*				
Yes	1.52 (1.18–1.96)	1.54 (1.19–1.98)	1.52 (1.12–2.05)	.9938
No	1.48 (1.32–1.65)	1.43 (1.28–1.61)	1.52 (1.32–1.74)	
*Hypertension*				
Yes	1.34 (1.13–1.58)	1.32 (1.11–1.56)	1.40 (1.15–1.70)	.3217
No	1.56 (1.37–1.78)	1.52 (1.33–1.74)	1.59 (1.35–1.86)	

Model 1 was adjusted for no covariates; Model 2 was adjusted for age, gender, race, marital status and education; Mode 3 = adjusted for all covariates except effect modifier.

CI = confidence interval, OR = odds ratio, TyG = triglyceride–glucose index.

## 4. Discussion

In a nationally representative sample of U.S. adults, a higher TyG index was independently associated with greater gallstone prevalence after comprehensive adjustment for demographic, lifestyle, metabolic, and clinical factors. The association was nonlinear, with a clear inflection at TyG = 8.97: below this threshold, odds of gallstones increased markedly with higher TyG, whereas above it the association plateaued. Results were stable across subgroups defined by sex, age, race/ethnicity, diabetes, and hypertension, with no evidence of effect modification.

The TyG index is a validated surrogate for IR that correlates with the hyperinsulinemic-euglycemic clamp and outperforms or complements Homeostatic Model Assessment of Insulin Resistance in several populations.^[14,15]^ Foundational studies by Simental-Mendía et al and Leay-kiaw Er et al supported its diagnostic utility and operational simplicity using routine laboratory measures.^[16,17]^ While research specifically linking TyG to gallstone disease remains sparse, prior work has connected IR to cholelithiasis risk. For instance, Méndez-Sánchez et al reported higher gallstone prevalence among individuals with IR quantified by Homeostatic Model Assessment of Insulin Resistance, suggesting a metabolic pathway to lithogenesis.^[16]^ Our analysis extends this literature by demonstrating, in U.S. adults, that an IR surrogate based on triglycerides and glucose is positively related to gallstones and by characterizing a threshold effect not previously delineated.

The association between IR and gallstone formation is biologically plausible through several interrelated pathways. IR promotes hepatic cholesterol overproduction by upregulating HMG-CoA reductase activity and increasing very-low-density lipoprotein output, which elevates biliary cholesterol secretion and drives cholesterol supersaturation of bile.^[18,19]^ Concurrently, hyperinsulinemia impairs cholecystokinin-mediated gallbladder contraction, leading to gallbladder hypomotility that favors bile stasis and crystal nucleation.^[20]^ IR is also linked to dysmetabolism of bile acids, with alterations in bile acid synthesis and enterohepatic circulation that can diminish the solubilizing capacity of bile.^[21,22]^ In addition, a triglyceride-rich milieu-captured by the TyG index-may reflect a lipoprotein profile that facilitates biliary cholesterol transport and saturation.^[23]^ Collectively, these mechanisms are consistent with prior reports of metabolic dysregulation in cholesterol gallstone pathogenesis and provide a rationale for the stronger association at lower-to-moderate TyG levels, with a saturation effect at higher levels where lithogenic pathways may already be maximally engaged.

Prior epidemiological studies have identified several independent risk factors for gallstones, including obesity, metabolic disturbances (dyslipidemia, diabetes, IR, metabolic syndrome), genetic susceptibility, and disrupted cholesterol homeostasis.^[24–27]^ Additional triggers that further increase risk include rapid weight loss and pregnancy.^[28–31]^ In our subgroup analyses, the association between TyG and gallstone prevalence was consistent across strata defined by race/ethnicity, diabetes, and hypertension, aligning with prior evidence in these groups. Moreover, we detected no effect modification by sex, age, race/ethnicity, hypertension, or diabetes (all *P* for interaction > .05), indicating that the relationship is stable across these demographic and clinical categories.

Strengths of this study include the use of NHANES, a nationally representative survey with standardized protocols and rigorous quality assurance, enhancing the reliability and generalizability of the findings. Comprehensive covariate adjustment and prespecified subgroup analyses further support the robustness of the association across diverse demographic and clinical strata. Several limitations should be noted. The cross-sectional design precludes causal inference. Gallstone status and several covariates were self-reported, which may introduce recall or misclassification bias. Finally, although the TyG index is a practical surrogate of IR, it is not a direct measure. While the analysis identifies a novel, nonlinear association between TyG and gallstone prevalence in U.S. adults, external validation and longitudinal studies are needed to confirm these results and establish temporality.

## 5. Conclusion

Our study found that higher TyG index were connected with increased prevalence of gallstones. Insulin resistance did not fully explain the findings. We hypothesized that treating and managing IR at a younger age may be beneficial in reducing the incidence of gallbladder stones. However, further large-scale prospective studies are still needed to clarify the precise causality of this relationship.

## Acknowledgments

The authors express their gratitude to the staff and participants of the NHANES study.

## Author contributions

**Conceptualization:** Xinlong Li.

**Data curation:** Jianan Tu.

**Formal analysis:** Jianan Tu.

**Investigation:** Jianan Tu.

**Software:** Jianan Tu.

**Writing – original draft:** Jianan Tu.

**Writing – review & editing:** Xinlong Li
